# Asking for Verbal Sexual Consent and Experiences of Sexual Violence and Sexual Behaviors Among High School Students — Youth Risk Behavior Survey, United States, 2023

**DOI:** 10.15585/mmwr.su7304a7

**Published:** 2024-10-10

**Authors:** Leigh E. Szucs, Sanjana Pampati, Kristen N. Jozkowski, Sarah DeGue, Catherine N. Rasberry, Anna W. Brittain, Casey Copen, Lexie Zimbelman, Sandra Leonard, Emily Young, Lindsay Trujillo

**Affiliations:** ^1^Division of Adolescent and School Health, National Center for Chronic Disease Prevention and Health Promotion, CDC, Atlanta, Georgia; ^2^Indiana University, Bloomington, Indiana; ^3^Division of Violence Prevention, National Center for Injury Prevention and Control, CDC, Atlanta, Georgia; ^4^Division of Reproductive Health, National Center for Chronic Disease Prevention and Health Promotion, CDC, Atlanta, Georgia; ^5^Division of STD Prevention, National Center for HIV, Viral Hepatitis, STD, and TB Prevention, CDC, Atlanta, Georgia

## Abstract

Adolescents’ sexual consent behaviors are critical for developing healthy sexual relationships and preventing experiences of sexual violence. This report uses 2023 Youth Risk Behavior Survey data to describe prevalence of asking for sexual consent verbally at last sexual contact among U.S. high school students. Differences in prevalence of asking for sexual consent verbally by sex, age, race and ethnicity, sexual identity, sex of sexual contacts, and gender identity were examined. Differences in asking for sexual consent verbally also were examined by experiences of sexual violence and sexual behaviors. Sex-stratified logistic regression analyses were performed to determine the association between asking for sexual consent verbally with experiences of sexual violence and sexual behaviors. In addition, data were analyzed using adjusted logistic regression models controlling for age, race and ethnicity, and sexual identity. Among high school students who reported ever having sexual contact, 79.8% reported asking for sexual consent verbally at last sexual contact. A lower percentage of female students (74.5%) reported asking for sexual consent verbally than male students (84.6%). In adjusted sex-stratified analyses, female students who asked for sexual consent verbally had higher prevalence of ever having had sexual intercourse. Male students who asked for sexual consent verbally had higher prevalence of ever having had sexual intercourse and being currently sexually active. Female and male students who asked for sexual consent verbally had higher prevalence of having first sexual intercourse before age 13 and using condoms. In addition, female students who asked for sexual consent verbally during last sexual intercourse had lower prevalence of using alcohol or drugs at last sexual intercourse. Public health researchers and practitioners, health care providers, schools, and youth-serving organizations can use these findings to better understand high school students’ verbal sexual consent, improve complex measurement of consent-seeking behaviors, and guide multicomponent sexual health and violence prevention efforts across various settings.

## Introduction

Sexual consent is a necessary foundation for all sexual activity and has implications for improving sexual health, including reducing sexual violence. Understanding sexual consent communication during adolescence is critical because many adolescents experiment with sexual behavior. In 2021, a total of 30% of U.S high school students reported ever having had sexual intercourse, with nearly one fourth having sex in the past 3 months (https://www.cdc.gov/healthyyouth/data/yrbs/yrbs_data_summary_and_trends.htm). Research examining consent behaviors among adolescents remains limited, but is essential, particularly because of associations between lack of consent and sexual violence.

To date, sexual consent research has primarily been conducted among college student populations (*1*). Evidence suggests college students hold positive attitudes toward affirmative consent (i.e., explicitly communicated, verbally or nonverbally) (*2*), despite more commonly using implicit and nonverbal cues to communicate consent (*1*,*2*). Differences by gender illustrate that, although implicit or nonverbal cues are more commonly used overall, college men report using verbal and explicit cues more frequently than college women, who frequently report using no response signals (i.e., letting sexual behaviors happen without resisting) to indicate consent (*3*). Notably, college students often are able to accurately interpret their partners’ cues; however, implicit and nonverbal cues are ambiguous and can be difficult to decipher, especially if persons have consumed alcohol or drugs before sexual activity (*3*). Significant gaps in knowledge about sexual consent behaviors remain, especially among adolescents (aged ≤18 years). The limited research available suggests adolescents also endorse positive attitudes toward explicit and affirmative sexual consent (*4*,*5*). However, research that examines how adolescents communicate consent is lacking, including whether differences exist in consent communication by sex, particularly whether adolescents use explicit, verbal consent.

To begin addressing these knowledge gaps, the 2023 national Youth Risk Behavior Survey (YRBS) included a new questionnaire item measuring whether U.S. high school students asked for sexual consent verbally at last sexual contact. This report presents the first national estimates for asking for sexual consent, verbally, among high school students. The report also examines whether the prevalence of asking for sexual consent verbally differed by experiences of sexual violence and sexual behaviors. The findings in this report can be used by public health researchers and practitioners, health care providers, schools, and youth-serving organizations to better understand high school students’ verbal sexual consent, improve complex measurement of consent-seeking behaviors, and guide multicomponent sexual health and violence prevention efforts across various settings.

## Methods

### Data Source

This report includes data from the 2023 YRBS (N = 20,103), a cross-sectional, school-based survey conducted biennially since 1991. Each survey year, CDC collects data from a nationally representative sample of public and private school students in grades 9–12 in the 50 U.S. states and the District of Columbia. Additional information about YRBS sampling, data collection, response rates, and processing is available in the overview report of this supplement (*6*). The prevalence estimates for asking for verbal sexual consent, experiences of sexual violence, and sexual behaviors for the study population overall and stratified by demographic characteristics are available at https://nccd.cdc.gov/Youthonline/App/Default.aspx. The full YRBS questionnaire, datasets, and documentation are available at https://www.cdc.gov/yrbs/index.html. Institutional reviews boards at CDC and ICF, the survey contractor, approved the protocol for YRBS. Data collection was conducted consistent with applicable Federal law and CDC policy.*

### Measures

YRBS questions, response options, and analytic coding are presented (Table 1). The primary outcome of interest was asking for sexual consent verbally at last sexual contact, determined by asking, “The last time you had sexual contact, did you ask for consent verbally?” (yes, no, or I have never had sexual contact). Because consent seeking is important in the context of adolescent sexual behaviors and violence, three experiences of sexual violence and eight sexual behaviors were examined. Demographic characteristics examined were sex (female or male); age (≤14, 15, 16, 17, or ≥18 years); race and ethnicity (American Indian or Alaska Native [AI/AN], Asian, Black or African American [Black], Native Hawaiian or other Pacific Islander [NH/OPI], White, Hispanic or Latino [Hispanic], or multiracial [selected >1 racial category]) (persons of Hispanic origin might be of any race but are categorized as Hispanic; all other racial groups are non-Hispanic); sexual identity (heterosexual, gay or lesbian, bisexual, questioning [I am not sure about my sexual identity/questioning], or describe identity in some other way [I describe my identity some other way]); sex of sexual contacts (opposite sex only, same sex only, or both sexes); and gender identity (cisgender, transgender, or questioning). The number of students who identified as NH/OPI was too small to include in analyses. Students who reported, “No, I am not transgender” to the gender identity variable were assumed to be cisgender. 

**TABLE 1:** Questions, response options, and analytic coding for asking for sexual consent verbally, experiences of sexual violence, and sexual behaviors — Youth Risk Behavior Survey, United States, 2023

Variable	Question	Response option	Analytic coding
Asking for sexual consent verbally	Consent is an agreement to do something or permission for something to happen. It can involve asking for consent, receiving consent, or giving consent. The last time you had sexual contact, did you ask for consent verbally?	Yes, no, or I have never had sexual contact	Yes versus no
**Experience of sexual violence**			
Experienced sexual violence by anyone during the past 12 months	During the past 12 months, how many times did anyone force you to do sexual things that you did not want to do? (Count such things as kissing, touching, or being physically forced to have sexual intercourse.)	0 times, 1 time, 2–3 times, 4–5 times, or ≥6 times	Yes (1 time, 2–3 times, 4–5 times, or ≥6 times) versus no (0 times)
Experienced sexual dating violence during the past 12 months	During the past 12 months, how many times did someone you were dating or going out with force you to do sexual things that you did not want to do? (Count such things as kissing, touching, or being physically forced to have sexual intercourse.)	I did not date or go out with anyone during the past 12 months, 0 times, 1 time, 2–3 times, 4–5 times, or ≥6 times	Yes (1 time, 2–3 times, 4–5 times, or ≥6 times) versus no (0 times)
Ever experienced forced sexual intercourse	Have you ever been physically forced to have sexual intercourse when you did not want to?	Yes or no	Yes versus no
**Sexual behavior**			
Ever had sexual intercourse	Have you ever had sexual intercourse?	I have never had sexual intercourse, yes, or no	Yes (yes) versus no (no or I have never had sexual intercourse)
First sexual intercourse before age 13 years	How old were you when you had sexual intercourse for the first time?	I have never had sexual intercourse, aged ≤11 years, 12 years, 13 years, 14 years, 15 years, 16 years, or ≥17 years	Yes (aged ≤11 years or 12 years) versus no (aged 13 years, 14 years, 15 years, 16 years, or ≥17 years)
Currently sexually active	During the past 3 months, with how many people did you have sexual intercourse?	I have never had sexual intercourse; I have had sexual intercourse, but not during the past 3 months; 1 person; 2 persons; 3 persons; 4 persons; 5 persons; or ≥6 persons	Yes (1 person, 2 persons, 3 persons, 4 persons, 5 persons, or ≥6 persons) versus no (I have never had sexual intercourse or I have had sexual intercourse, but not during the past 3 months)
Alcohol or drug use during last sexual intercourse	Did you drink alcohol or use drugs before you had sexual intercourse the last time?	I have never had sexual intercourse, yes, or no	Yes versus no
Condom use during last sexual intercourse	The last time you had sexual intercourse, did you or your partner use a condom?	I have never had sexual intercourse, yes, or no	Yes versus no
Highly or moderately effective hormonal contraceptive method use during last sexual intercourse	The last time you had sexual intercourse with an opposite-sex partner, what one method did you or your partner use to prevent pregnancy?	I have never had sexual intercourse with an opposite-sex partner; no method was used to prevent pregnancy; birth control pills (not including emergency contraception such as Plan B or the morning after pill); condoms; an IUD (such as Mirena or ParaGard) or implant (such as Implanon or Nexplanon); a shot (such as Depo-Provera), patch (such as Ortho Evra), or birth control ring (such as NuvaRing); withdrawal or some other method; or not sure	Yes (birth control pills; an IUD or implant; or a shot, patch, or birth control ring) versus no (no method was used to prevent pregnancy, condom, withdrawal or some other method, or not sure)
Condoms as primary contraceptive method during last sexual intercourse	The last time you had sexual intercourse with an opposite-sex partner, what one method did you or your partner use to prevent pregnancy?	I have never had sexual intercourse with an opposite-sex partner; no method was used to prevent pregnancy; birth control pills (not including emergency contraception such as Plan B or the morning after pill); condoms; an IUD (such as Mirena or Paragard) or implant (such as Implanon or Nexplanon); a shot (such as Depo-Provera), patch (such as Ortho Evra), or birth control ring (such as NuvaRing); withdrawal or some other method; or not sure	Yes (condoms) versus no (birth control pills; an IUD or implant; a shot, patch, or birth control ring; no method was used to prevent pregnancy; withdrawal or some other method; or not sure)
Receipt of STI testing during the past 12 months	During the past 12 months, have you been tested for a sexually transmitted disease other than HIV, such as chlamydia or gonorrhea?	Yes, no, or not sure	Yes versus no

**Abbreviations:** IUD = intrauterine device; STI = sexually transmitted infection.

### Analysis

The analytic sample was restricted to those who ever had sexual contact (n = 5,492). For analyses that involved the variable of experienced sexual dating violence, the sample was further restricted to those who reported going on a date or going out with anyone in the past 12 months. For analyses that involved the variables of first sexual intercourse before age 13 years, alcohol or drug use during last sexual intercourse, and condom use during last sexual intercourse, the sample was further restricted to those who ever had sexual intercourse. For analyses examining primary contraceptive method used to prevent pregnancy during last sexual intercourse, the sample was further restricted to those who ever had sexual intercourse with an opposite-sex partner.

All prevalence estimates and measures of association used Taylor series linearization. Weighted prevalences and 95% CIs of asking for sexual consent verbally at last sexual contact by demographic characteristics are presented for the total study population and stratified by sex. Differences in prevalence of asking for sexual consent verbally by age, race and ethnicity, sexual identity, and sex of sexual contacts were examined using pairwise *t*-tests among female and male students. Overall, among students in the analytic sample, prevalence of asking for sexual consent verbally was examined by gender identity. Sex-stratified prevalences and 95% CIs of asking for sexual consent verbally by each experience of sexual violence and behavior are presented. Pairwise *t*-test analysis was used to examine whether the sex-stratified prevalence of asking for sexual consent verbally at last sexual contact differed by each experience of sexual violence and sexual behavior. Differences assessed by *t*-tests were considered statistically significant for p values <0.05. Sex-stratified logistic regression analyses were performed to determine the association between asking for sexual consent verbally with experiences of sexual violence and sexual behaviors. Adjusted prevalence ratios were calculated using logistic regression with predicted marginals. Adjusted models included age, race and ethnicity, and sexual identity. Model-adjusted–risk differences were assessed through pairwise comparisons of students who asked for verbal sexual consent versus those who did not. Statistical significance was determined by whether the 95% CI of the adjusted prevalence ratio did not include 1.0 or if the model-adjusted risk difference p value was <0.05. Prevalence estimates with denominators <30 were considered statistically unreliable and therefore suppressed (*7*). Analyses were conducted using SAS-callable SUDAAN (version 11.0.3; RTI International) to account for the complex sampling design and weighting.

## Results

Among U.S. high school students who had sexual contact (n = 5,492), 79.8% reported asking for sexual consent verbally at last sexual contact (Table 2). Female students were less likely to ask for sexual consent verbally than male students (74.5% versus 84.6%). Among females, students aged ≥18 years were less likely (66.4%) to ask for sexual consent verbally than students aged 17 years (78.0%) and 16 years (76.5%). Among females, Asian students were more likely to ask for sexual consent verbally (92.3%) than their Hispanic (75.1%), multiracial (75.0%), White (74.0%), Black (73.2%), and AI/AN (72.1%) peers. Female students who had same-sex–only sexual contacts were more likely to ask for sexual consent verbally (85.9%) than female students who reported opposite-sex–only contacts (73.4%) or both sexes (76.0%). Among males, Black students were less likely to ask for sexual consent verbally (76.0%) than Hispanic (87.6%) and White students (85.3%). Male students identifying as bisexual had a higher prevalence of asking for sexual consent verbally (94.2%) than male students identifying as heterosexual (85.2%) or questioning (65.8%). Male students identifying as gay also were more likely than those questioning their sexual identity to ask for sexual consent verbally (86.6% versus 65.8%). Male students who reported sexual contact with both sexes were more likely to ask for sexual consent verbally than male students who had same-sex–only contacts (89.1% versus 77.8%).

**TABLE 2:** Prevalence of asking for sexual consent verbally at last sexual contact among high school students who ever had sexual contact, by demographic characteristics and sex — Youth Risk Behavior Survey, United States, 2023*

Characteristic	Asked for sexual consent verbally at last sexual contact^†^		
	Total^§ ^% (95% CI)^¶^	Female % (95% CI)^¶^	Male % (95% CI)^¶^
	79.8 (77.8–81.7)	74.5 (71.6–77.1)**	84.6 (82.0–86.9)
**Age, yrs**			
≤14	**78.6 (73.1–83.2)**	70.6 (61.4–78.4)	86.0 (78.0–91.4)
15	**80.8 (77.2–84.0)**	74.3 (67.8–80.0)	86.5 (82.4–89.9)
16	**81.8 (79.3–84.1)**	76.5 (72.2–80.4)^††^	86.0 (82.2–89.1)
17	**80.5 (77.7–83.0)**	78.0 (73.8–81.7)^§§^	82.9 (78.8–86.4)
≥18	**74.9 (70.0–79.3)**	66.4 (57.5–74.3)	82.3 (77.5–86.2)
**Race and ethnicity** ^¶¶^			
American Indian or Alaska Native	**78.2 (67.6–86.0)**	72.1 (53.3–85.4)***	87.3 (73.1–94.5)
Asian	**89.9 (82.8–94.3)**	92.3 (83.1–96.7)^†††,§§§,¶¶¶,^****	88.1 (73.9–95.1)
Black or African American	**74.6 (66.9–81.0)**	73.2 (63.7–80.9)	76.0 (67.4–82.8)^††††,§§§§^
White	**79.9 (77.2–82.4)**	74.0 (69.4–78.0)	85.3 (82.1–88.0)
Hispanic or Latino	**81.5 (78.6–84.1)**	75.1 (71.5–78.4)	87.6 (82.8–91.2)
Multiracial	**81.7 (76.2–86.2)**	75.0 (67.0–81.6)	86.3 (77.9–91.9)
**Sexual identity**			
Heterosexual (straight)	**81.1 (78.6–83.4)**	74.4 (69.9–78.5)	85.2 (82.6–87.4)^¶¶¶¶^
Lesbian or gay	**80.9 (72.6–87.2)**	76.9 (65.6–85.3)	86.6 (75.6–93.1)*****
Bisexual	**80.0 (75.3–84.0)**	76.7 (72.0–80.8)	94.2 (85.8–97.8)^†††††^
Identify some other way^§§§§§^	**72.8 (60.9–82.2)**	71.3 (58.2–81.7)	77.6 (54.2–91.0)
Questioning	**73.1 (63.5–81.0)**	75.0 (64.6–83.1)	65.8 (44.9–82.0)
**Sex of sexual contacts**			
Opposite sex only	**80.1 (77.7–82.3)**	73.4 (69.5–77.0)^¶¶¶¶¶^	84.9 (82.1–87.4)
Same sex only	**82.8 (75.6–88.3)**	85.9 (76.8–91.8)******	77.8 (68.0–85.3)^††††††^
Both sexes	**78.5 (72.9–83.2)**	76.0 (69.7–81.4)	89.1 (78.7–94.7)
**Gender identity**			
Cisgender	**80.2 (78.3–82.0)**	—	—
Transgender	**76.6 (61.9–86.8)**	—	—
Questioning	**76.8 (61.7–87.2)**	—	—

**Abbreviation:** YRBS = Youth Risk Behavior Survey.

* N = 20,103 respondents. The total number of students answering each question varied. Data might be missing because 1) the question did not appear in that student’s questionnaire, 2) the student did not answer the question, or 3) the response was set to missing because of an out-of-range response or logical inconsistency. Percentages in each category are calculated on the known data.

^†^ Prevalence of asking for sexual consent was among those who ever had sexual contact.

^§^ The total (N = 5,492 [2,662 females and 2,806 males]) is unweighted.

^¶^ Weighted. Due to missing data, the total for each group will not add to the total reported.

** Male students significantly differed from female students on the overall prevalence of asking for sexual consent verbally based on *t*-test analysis with Taylor series linearization (p<0.05).

^††^ Among female students, those aged 16 years significantly differed from students aged ≥18 years based on *t*-test analysis with Taylor series linearization (p<0.05).

^§§^ Among female students, those aged 17 years significantly differed from students aged ≥18 years based on *t*-test analysis with Taylor series linearization (p<0.05).

^¶¶^ Persons of Hispanic or Latino origin might be of any race but are categorized as Hispanic; all racial groups are non-Hispanic. Due to a small sample size, Native Hawaiian or Pacific Islander estimates were suppressed and not reported.

*** Among female students, American Indian or Alaska Native students significantly differed from Asian students based on *t*-test analysis with Taylor series linearization (p<0.05).

^†††^ Among female students, Asian students significantly differed from Black or African American students based on *t*-test analysis with Taylor series linearization (p<0.05).

^§§§^ Among female students, Asian students significantly differed from White students based on *t*-test analysis with Taylor series linearization (p<0.05).

^¶¶¶^ Among female students, Asian students significantly differed from Hispanic or Latino students based on *t*-test analysis with Taylor series linearization (p<0.05).

**** Among female students, Asian students significantly differed from multiracial students based on *t*-test analysis with Taylor series linearization (p<0.05).

^††††^ Among male students, Black or African American students significantly differed from White students based on *t*-test analysis with Taylor series linearization (p<0.05).

^§§§§^ Among male students, Black or African American students significantly differed from Hispanic or Latino students based on *t*-test analysis with Taylor series linearization (p<0.05).

^¶¶¶¶^ Among male students, heterosexual students significantly differed from bisexual students based on *t*-test analysis with Taylor series linearization (p<0.05).

***** Among male students, gay students significantly differed from questioning students based on *t*-test analysis with Taylor series linearization (p<0.05).

^†††††^ Among male students, bisexual students significantly differed from questioning students based on *t*-test analysis with Taylor series linearization (p<0.05).

^§§§§§^ Based on YRBS question, students who choose the response “I describe my sexual identity some other way” when referencing their sexual identity.

^¶¶¶¶¶^ Among female students, students who had sexual contact with the opposite sex only significantly differed from students who had sexual contact with the same sex only based on *t*-test analysis with Taylor series linearization (p<0.05).

****** Among female students, students who had sexual contact with the same sex only significantly differed from students who had sexual contact with both sexes based on *t*-test analysis with Taylor series linearization (p<0.05).

^††††††^ Among male students, students who had sexual contact with the same sex only significantly differed from students who had sexual contact with both sexes based on *t*-test analysis with Taylor series linearization (p<0.05).

No differences were found in the sex-stratified prevalence of asking for sexual consent verbally by any experiences of sexual violence (Table 3). Students who ever had sexual intercourse versus not (female students: 76.3% and 70.6%, respectively; male students: 86.8% and 80.2%, respectively) or were currently sexually active versus not (female students: 77.2% and 72.1%, respectively; male students: 87.2% and 82.8%, respectively) had a higher prevalence of having asked for sexual consent verbally at last sexual contact (Table 3). Male students who reported first sexual intercourse before age 13 years reported lower prevalence of asking for sexual consent verbally than those who had their first sexual intercourse after age 13 years (74.7% versus 85.4%). Students who used a condom during last sexual intercourse versus not had a higher prevalence of having asked for sexual consent verbally at last sexual contact (female students: 80.5% and 72.3%, respectively; male students: 90.4% and 80.9%, respectively) (Table 3). For male students, those who reported using condoms as their or their partner’s primary contraceptive method during last sexual intercourse had higher prevalence of asking for sexual consent verbally than those who did not (90.4% versus 83.2%) (Table 3).

**TABLE 3:** Sex-stratified prevalence of asking for sexual consent verbally at last sexual contact by experiences of sexual violence and sexual behaviors among high school students — Youth Risk Behavior Survey, United States, 2023*

Experience of sexual violence or sexual behavior^†^	Asked for sexual consent verbally at last sexual contact^§^	
	Female % (95% CI)^¶^	Male % (95% CI)^¶^
**Sexual violence**		
Experienced sexual violence by anyone during the past 12 months**		
Yes	71.6 (67.6–75.4)	80.4 (71.0–87.3)
No	75.4 (72.1–78.4)	85.2 (82.5–87.5)
Experienced sexual dating violence during the past 12 months^††,§§^		
Yes	76.3 (70.8–81.1)	85.7 (75.3–92.1)
No	75.1 (72.2–77.9)	85.1 (82.4–87.5)
Ever experienced forced sex^¶¶^		
Yes	70.7 (64.3–76.4)	82.8 (74.1–89.0)
No	75.8 (73.0–78.4)	84.8 (82.0–87.2)
**Sexual behavior**		
Ever sexual intercourse***^,†††,§§§^		
Yes	76.3 (72.7–79.5)	86.8 (84.3–88.9)
No	70.6 (66.1–74.7)	80.2 (75.5–84.2)
First sexual intercourse before age 13^†††,¶¶¶^		
Yes	56.7 (44.8–67.9)	74.7 (61.3–84.6)
No	75.3 (72.5–78.0)	85.4 (83.0–87.5)
Currently sexually active^†††,§§§,^****		
Yes	77.2 (72.9–81.0)	87.2 (84.2–89.7)
No	72.1 (68.5–75.4)	82.8 (78.9–86.1)
Alcohol or drug use during last sexual intercourse^††††,§§§§^		
Yes	69.6 (59.7–78.0)	80.8 (73.1–86.6)
No	77.7 (74.4–80.6)	87.8 (84.9–90.2)
Condom use during last sexual intercourse^†††,§§§,††††,¶¶¶¶^		
Yes	80.5 (75.8–84.5)	90.4 (87.4–92.8)
No	72.3 (66.3–77.7)	80.9 (76.3–84.7)
Highly or moderately effective hormonal contraceptive method use during last sexual intercourse*****		
Yes	75.4 (69.5–80.5)	85.5 (77.6–90.9)
No	76.7 (69.9–82.4)	87.8 (84.4–90.6)
Condoms as primary contraceptive method during last sexual intercourse^§§§,^*****^,§§§§§^		
Yes	79.1 (71.7–84.9)	90.4 (87.0–93.0)
No	72.6 (67.5–77.2)	83.2 (78.6–86.9)
Received STI testing during the past 12 months^¶¶¶¶¶^		
Yes	73.8 (63.3–82.2)	81.3 (68.5–89.7)
No	74.4 (71.6–77.0)	85.1 (82.3–87.5)

**Abbreviation:** STI = sexually transmitted infection.

* N = 20,103 respondents. The total number of students answering each question varied. Data might be missing because 1) the question did not appear in that student’s questionnaire, 2) the student did not answer the question, or 3) the response was set to missing because of an out-of-range response or logical inconsistency. Percentages in each category are calculated on the known data.

^†^ Unweighted counts indicating denominators for females and males for each experience of sexual violence and sexual behavior. The total number of students answering each question varied. Percentages in each category were calculated on the known data.

^§^ Prevalence of asking for sexual consent verbally was among those who ever had sexual contact. The total (N = 5,492 [2,662 females and 2,806 males]) is unweighted.

^¶^ Weighted.

** Includes 2,551 female and 2,755 male respondents.

^††^ Among students who dated or went out with someone during the 12 months before the survey.

^§§^ Includes 2,482 female and 2,709 male respondents.

^¶¶^ Includes 2,615 female and 2,770 male respondents.

*** Includes 2,646 female and 2,780 male respondents.

^†††^ Pairwise *t*-tests were conducted to determine whether prevalence of asking for sexual consent verbally differed by each experience of sexual violence and sexual behavior among females. Differences are statistically significant at p<0.05.

^§§§^ Pairwise *t*-tests were conducted to determine whether prevalence of asking for sexual consent verbally differed by each experience of sexual violence and sexual behavior among males. Differences are statistically significant at p<0.05.

^¶¶¶^ Includes 2,626 female and 2,767 male respondents.

**** Includes 2,527 female and 2,675 male respondents.

^††††^ Among students who ever had sexual intercourse.

^§§§§^ Includes 1,873 female and 2,025 male respondents.

^¶¶¶¶^ Includes 1,794 female and 1,962 male respondents.

***** For use of highly or moderately effective hormonal contraceptive method and condom as primary contraceptive method, sample was restricted to those who ever had sexual intercourse with an opposite-sex partner.

^†††††^ Includes 1,148 female and 1,198 male respondents.

^§§§§§^ Includes 1,668 female and 1,882 male respondents.

^¶¶¶¶¶^ Includes 2,600 female and 2,724 male respondents.

In analyses adjusted for age, race and ethnicity, and sexual identity, female students who asked for sexual consent verbally had higher prevalence of ever having had sexual intercourse, higher prevalence of using condoms, lower prevalence of first sexual intercourse before age 13, and lower prevalence of using alcohol or drugs during last sexual intercourse (Table 4). Male students who asked for sexual consent verbally had higher prevalence of ever having had sexual intercourse, and being currently sexually active, and lower prevalence of first sexual intercourse before age 13. Male students who asked for sexual consent verbally had higher prevalence of using a condom during last sexual intercourse and using a condom as their or their partner’s primary contraceptive method to prevent pregnancy during last sexual intercourse with an opposite-sex partner than those who did not ask for sexual consent (Table 4).

**TABLE 4:** Associations between asking for sexual consent verbally with experiences of sexual violence and sexual behaviors among high school students, by sex — Youth Risk Behavior Survey, United States, 2023*

Experience of sexual violence or sexual behavior	Asked for sexual consent verbally at last sexual contact^†^	
	Female aPR (95% CI)^§^	Male aPR (95% CI)^§^
**Experience of sexual violence**		
Experienced sexual violence by anyone during the past 12 months	0.87 (0.74–1.01)	0.76 (0.49–1.17)
Experienced sexual dating violence during the past 12 months^¶^	1.02 (0.76–1.37)	1.01 (0.54–1.90)
Ever experienced forced sexual intercourse	0.81 (0.65–1.01)	0.81 (0.46–1.42)
**Sexual behavior**		
Ever sexual intercourse	1.10 (1.00–1.22)**	1.20 (1.07–1.34)**^,††^
First sexual intercourse before age 13^§§^	0.42 (0.27–0.67)**^,††^	0.47 (0.28–0.79)**^,††^
Currently sexually active	1.14 (0.98–1.31)	1.26 (1.02–1.55)**^,††^
Alcohol or drug use during last sexual intercourse^§§^	0.67 (0.47–0.95)**^,††^	0.68 (0.43–1.05)
Condom use during last sexual intercourse^§§^	1.34 (1.04–1.74)**^,††^	1.43 (1.11–1.84)**^,††^
Highly or moderately effective hormonal contraceptive method use during last sexual intercourse^¶¶^	1.02 (0.77–1.34)	0.91 (0.58–1.42)
Condoms as primary contraceptive method during last sexual intercourse^¶¶^	1.31 (0.95–1.79)	1.43 (1.05–1.93)**^,††^
Received STI testing during the past 12 months	1.01 (0.68–1.49)	0.97 (0.45–2.07)

**Abbreviations:** aPR = adjusted prevalence ratio; STI = sexually transmitted infection.

* N = 20,103 respondents. The total number of students answering each question varied. Data might be missing because: 1) the question did not appear in that student’s questionnaire, 2) the student did not answer the question, or 3) the response was set to missing because of an out-of-range response or logical inconsistency. Percentages in each category are calculated on the known data.

^†^ Prevalence ratio of experiences of sexual violence and sexual behaviors comparing students who asked for sexual consent verbally to those who did not ask for sexual consent verbally, among students who ever had sexual contact.

^§^ aPR adjusted for age, race and ethnicity, and sexual identity.

^¶^ Among students who dated or went out with someone during the 12 months before the survey.

** Model-adjusted risk differences were assessed through pairwise comparisons of students who asked for verbal sexual consent versus those who did not. Differences were considered statistically significant at p<0.05.

^††^ aPRs were considered significant if the 95% CI did not cross the null value of 1.00.

^§§^Among students who ever had sexual intercourse.

^¶¶^ Among students who ever had sexual intercourse with an opposite-sex partner.

## Discussion

Findings from this report indicate that, in 2023, among those reporting ever having had sexual contact, approximately eight in 10 U.S. high school students asked for sexual consent verbally at last sexual contact. Reports of asking for sexual consent verbally among high school students were substantially higher in this study than among most college student samples in previous research (*1*–*3*,*8*). A few possible explanations exist for this observation. Conceptually, adolescents and young adults might be more attuned to the significance of consent through increased public discourse and intervention, resulting in potentially greater use of verbal consent, and all age groups are likely influenced by some desirability bias leading to any increased reporting of use of verbal consent on surveys. Methodologically, measurement of consent behaviors among college students often comprises questions that ask participants to describe consent cues they have used or select from an array of response options that include explicit and implicit verbal cues, among others (*8*). This difference in measures may also explain some discrepancies in the responses between high school and college students.

Although previous research suggested links between sexual violence victimization and decreased sexual communication (*7*), this report found no significant differences in the prevalence of asking for sexual consent verbally between those who had experienced sexual violence by anyone, sexual dating violence, or forced sex compared with those who had not. This finding suggests that consent-seeking behaviors were not associated, positively or negatively, with a history of experiencing sexual violence. Because of high rates of reporting for consent seeking in this report, limited variability might have reduced the ability to detect a relation between asking for sexual consent verbally and experiences of sexual violence. Furthermore, YRBS measures self-reported experiences of sexual violence victimization but not perpetration experiences. A history of sexual violence perpetration that involves nonconsensual sexual contact by definition might be associated with not asking someone for sexual consent at last sexual contact. Additional research examining a wider range of sexual violence and consent experiences (e.g., perpetration, explicit and implicit verbal cues, and explicit and implicit nonverbal cues) is needed to better understand the potential effects of experiencing sexual violence victimization on consent behaviors.

Overall, female students were less likely to ask for sexual consent verbally than male students. This difference might reflect more frequent sexual initiation by male compared with female students (*9*), activating consent that might be shaped by social or cultural sexual scripts and expectations (*10*). Furthermore, traditional gender-based roles and power dynamics, which suggest that males, and not females, are responsible for initiating sexual behavior and gaining consent, might influence actions (*11*). Differences by sex also align with previous research citing sex discordance in communicating and interpreting sexual consent among females and males (*11*). Finally, considering the finding of age-related differences, particularly among females, future research should examine individual (e.g., attitudes and internalized gender roles) and relationship (e.g., length of time) factors (*12*) on consent-seeking behaviors across early versus later adolescent developmental periods.

Certain differences were found in asking for sexual consent verbally by race and ethnicity and sexual identity status. Among females, prevalence was highest among Asian students; among males, prevalence was lowest among Black students. Bisexual male students were more likely to ask for sexual consent verbally than heterosexual peers, and gay male students were more likely to ask for sexual consent verbally than those questioning their sexual identity. These findings are consistent with qualitative studies describing sexual minority adolescents and young adults’ report of self-efficacy and comfort with sexual consent communication (*13*). Together, the findings in this report and others (*14*), emphasize the need for a more nuanced examination of how consent intersects with multiple identities and sexual activity norms and expectations. Sexual consent research primarily comprises White, heterosexual, and cisgender college student samples (*1*–*3*,*7*), which is insufficient for understanding consent among diverse populations (*13*,*14*). This report’s findings begin to address this gap by presenting consent behavior among adolescents of various racial, ethnic, sexual, and gender identities in a nationally representative population-based sample. Future studies to explore sexual consent among adolescents across multiple intersecting identities could help address remaining gaps.

Adolescents who reported asking for sexual consent verbally reported higher prevalence of ever having had sexual intercourse, lower prevalence of first sexual intercourse before age 13 years, and higher prevalence of being currently sexually active (males only). Such associations might be due, in part, to the fact that explicit verbal consent is commonly used in the context of sexual intercourse (*3*) and, as adolescents have sexual intercourse, opportunities to ask for consent might be needed. Asking for sexual consent verbally was associated with lower prevalence of using alcohol or drugs during last sexual intercourse among female students, which is critical given robust evidence showing disproportionate experiences of substance use–related, nonconsensual sexual activity among college females (*15*). Studies note male students’ frequent use of condoms, including as the primary contraceptive method during last sexual intercourse, and the association of frequent condom use with asking for sexual consent verbally could reflect that males might be more inclined to initiate consent through discussions of condom use, which is a common consent cue reported by young persons (*3*,*8*). In light of trending declines in condom use (https://www.cdc.gov/healthyyouth/data/yrbs/yrbs_data_summary_and_trends.htm) and high rates of sexually transmitted infections (STIs) among adolescents and young adults (https://www.cdc.gov/std/statistics/2022/slides/2022-STI-Surveillance-Adolescents-and-Young-Adults.pptx), this higher prevalence (80.5% and 90.4%) of condom use for any reason among female and male students who asked for sexual consent verbally is promising. Taken together, such findings might suggest an association between verbal consent and protective sexual behaviors, with verbal consent potentially reflecting a protective behavior as well. Comprehensive approaches to sexual health promotion and violence prevention could be leveraged to increase protective sexual behaviors including asking for verbal consent, using condoms, and avoiding alcohol and drug use before and during sexual activity. Furthermore, because of the lack of association between asking for sexual consent verbally and use of a moderately or highly effective contraceptive method among female students, future investigations could consider factors influencing contraceptive decision-making and the role consent plays for female students and their sexual partners.

## Limitations

General limitations of the YRBS are available in the overview report of this supplement (*6*). The findings in this report are subject to at least six additional limitations. First, the reliance on a single item to measure asking for sexual consent verbally captures only one dimension of consent; it lacks depth in consistently examining other aspects (e.g., diversity in cues being asked, withdrawing or negotiating consent between partners, or for specific sexual acts). Second, asking for sexual consent verbally was presented in relation to last “sexual contact” and did not define specific behaviors (e.g., penetrative intercourse, hand-to-genital contact, and kissing), influencing interpretability. Sexual contact was possibly understood as distinct from other sexual behaviors measured on YRBS. Third, variation in recall periods across different behavioral measures (e.g., last sexual contact, last sexual intercourse, or the past 12 months) introduces complexities in understanding the context in which sexual consent was sought and should be considered. However, this report presents the first national estimates for asking for sexual consent verbally as an important first step in understanding adolescent consent-seeking behavior. Future studies could employ multidimensional scales to capture the complexity and nuances of consent behaviors and experiences among adolescents (*3*,*16*). Fourth, sexual violence was measured with three items and might not encompass the full range of violence experienced by students. Fifth, disentangling condom use for pregnancy prevention versus STI and HIV prevention is not feasible. Although YRBS measures condom use as a primary method for pregnancy prevention (https://www.cdc.gov/healthyyouth/data/yrbs/questionnaires.htm), condom use for STI and HIV prevention is not explicitly measured and might differ by consent endorsement. Finally, because this analysis included only students who have ever had sexual contact, certain estimates (e.g., student groups stratified by race and ethnicity) with a denominator <30 were considered statistically unreliable and therefore suppressed (*7*).

## Future Directions

These findings point to next steps for sexual consent research and practice to support adolescent sexual health education and violence prevention. Most importantly, future studies might be strengthened by employing comprehensive, multidimensional scales to determine complexity of consent behaviors. Guided by valid and reliable consent scales (*4*,*16*), public health surveillance and research measurement could be expanded to reflect the breadth and depth of consent in context of adolescents’ overlapping identities, experiences of sexual violence, and the range of sexual contacts including with whom, how, and when adolescents engage in risky and protective sexual behaviors. More refined sexual consent measurement would contribute substantially to emerging evidence with adolescents (*4*,*5*,*12*) and more robust studies with young adult and college student samples (*1*–*3*).

Multicomponent prevention efforts also are needed to better understand and address the development of sexual communication skills and social norms that support consent behaviors among youths and young adults. Schools and youth-serving organizations are uniquely positioned to address consent knowledge, self-efficacy, and behaviors through skills-focused programming (*17*). CDC’s Health Education Curriculum Analysis Tool (https://www.cdc.gov/healthyyouth/hecat/pdf/2021/hecat_module_sh.pdf) could help identify developmentally recommended, culturally responsive, and inclusive curricula across grades K–12. Consent education also is included in many sexual and teen dating violence prevention approaches. CDC’s comprehensive dating violence prevention model, Dating Matters, teaches middle school students about healthy relationship skills, including consent communication, and has been found to reduce sexual violence, sexual harassment, and dating violence behaviors (https://vetoviolence.cdc.gov/apps/dating-matters-toolkit/). Health care providers can talk with adolescents about sexual consent and work to recognize occurrences of nonconsensual sexual activity, providing indicated care and referrals (*18*). Public health professionals also can help parents build skills for effectively and consistently communicating with their adolescents about consent, sexual behaviors (including condom negotiation), and preventing violence, because sexual communication is linked to increased parent-child communication about consent for both mothers and fathers (*19*).

## Conclusion

Adolescents’ awareness of and communication about sexual consent are critical components for preventing and reducing experiences of violence and promoting healthy sexuality. YRBS nationally representative data indicate asking for sexual consent verbally among U.S. high school students was high but varied by demographic characteristics and engagement in sexual behaviors. Differences were detected in verbal consent among female and male students, and in association with sexual intercourse, being currently sexually active (males only), condom use, older age at first sexual intercourse, and nonuse of alcohol and drugs during last sexual intercourse (females only). Future work to measure the complexity and multidimensional nature of consent behavior is needed and might guide prevention efforts addressing sexual health and violence prevention across settings. Schools and youth-serving organizations, as well as health care professionals and parents, have an important role in bolstering adolescents’ communication, understanding, and use of consent with partners; participating in informed sexual decision-making; and reducing negative sexual health and violence outcomes.

## References

[1] Muehlenhard CL, Humphreys TP, Jozkowski KN, Peterson ZD. The complexities of sexual consent among college students: a conceptual and empirical review. J Sex Res 2016;53:457–87. 10.1080/00224499.2016.1146651 27044475

[2] Willis M, Blunt-Vinti HD, Jozkowski KN. Associations between internal and external sexual consent in a diverse national sample of women. Pers Individ Dif 2019;149:37–45. 10.1016/j.paid.2019.05.029

[3] Jozkowski KN, Sanders S, Peterson ZD, Dennis B, Reece M. Consenting to sexual activity: the development and psychometric assessment of dual measures of consent. Arch Sex Behav 2014;43:437–50. 10.1007/s10508-013-0225-7 24452630

[4] Javidi H, Maheux AJ, Widman L, Kamke K, Choukas-Bradley S, Peterson ZD. Understanding adolescents’ attitudes toward affirmative consent. J Sex Res 2020;57:1100–7. 10.1080/00224499.2019.1711009 31940226 PMC7363507

[5] Righi MK, Bogen KW, Kuo C, Orchowski LM. A qualitative analysis of beliefs about sexual consent among high school students. J Interpers Violence 2021;36:NP8290–316. 10.1177/0886260519842855 30973037

[6] Brener ND, Mpofu JJ, Krause KH, Overview and methods for the Youth Risk Behavior Surveillance System—United States, 2023. In: Youth Risk Behavior Surveillance—United States, 2023. MMWR Suppl 2024;73(No. Suppl-4):1–12. 10.15585/mmwr.su7304a1 PMC11559678 39378301

[7] Vitek KN, Yeater EA. The association between a history of sexual violence and romantic relationship functioning: a systematic review. Trauma Violence Abuse 2021;22:1221–32. 10.1177/1524838020915615 32242504

[8] Jozkowski KN, Peterson ZD, Sanders SA, Dennis B, Reece M. Gender differences in heterosexual college students’ conceptualizations and indicators of sexual consent: implications for contemporary sexual assault prevention education. J Sex Res 2014;51:904–16. 10.1080/00224499.2013.792326 23919322

[9] Haydon AA, Herring AH, Prinstein MJ, Halpern CT. Beyond age at first sex: patterns of emerging sexual behavior in adolescence and young adulthood. J Adolesc Health 2012;50:456–63. 10.1016/j.jadohealth.2011.09.006 22525108 PMC3336094

[10] Healy Cullen S, O’Rourke T, O’Higgins S, Using communication stories to explore how young people draw on sexual scripts when making sense of sexual consent. Sex Cult 2023;27:1556–77. 10.1007/s12119-023-10078-y

[11] Richards MJ, Bogart A, Sheeder J. Communication and interpretation of sexual consent and refusal in adolescents and young adults. J Adolesc Health 2022;70:915–21. 10.1016/j.jadohealth.2021.12.013 35165034

[12] Javidi H, Anderson P, Walsh-Buhi E, Coyle K, Chen X. Exploring the influence of romantic relationship communication on adolescents’ self-efficacy to ask for sexual consent. J Sex Res 2024;61:1–9. 10.1080/00224499.2024.2306475 38295004

[13] de Heer B, Brown M, Cheney J. Sexual consent and communication among the sexual minoritized: the role of heteronormative sex education, trauma, and dual identities. Fem Criminol 2021;16:701–21. 10.1177/15570851211034560

[14] McKenna JL, Roemer L, Orsillo SM. Predictors of sexual consent attitudes, beliefs, and behaviors among sexual minority cisgender and nonbinary young adults. Sex Roles 2021;85:391–404. 10.1007/s11199-021-01226-5

[15] Griffin KW, Lindley LL, Cooper Russell E, Mudd T, Williams C, Botvin GJ. Sexual violence and substance use among first-year university women: differences by sexual minority status. Int J Environ Res Public Health 2022;19:10100. 10.3390/ijerph191610100 36011735 PMC9407960

[16] Glace AM, Zatkin JG, Kaufman KL. Moving toward a new model of sexual consent: the development of the process-based consent scale. Violence Against Women 2021;27:2424–50. 10.1177/1077801220952159 32865133

[17] Burton O, Rawstorne P, Watchirs-Smith L, Nathan S, Carter A. Teaching sexual consent to young people in education settings: a narrative systematic review. Sex Educ 2021;23:18–34. 10.1080/14681811.2021.2018676

[18] Society for Adolescent Health and Medicine. Promoting sexual consent principles in the sexual and reproductive health care of adolescents and young adults. J Adolesc Health 2023;73:205–9. 10.1016/j.jadohealth.2023.04.002 37149808

[19] Padilla-Walker LM, McLean R, Ogles B, Pollard B. How do parents teach “no means no”? An exploration of how sexual consent beliefs are socialized during adolescence. J Sex Res 2020;57:1122–33. 10.1080/00224499.2020.1792397 32723188

